# Differential redox sensitivity of tRNA dihydrouridylation

**DOI:** 10.1093/nar/gkae964

**Published:** 2024-10-26

**Authors:** Lea-Marie Kilz, Simone Zimmermann, Virginie Marchand, Valérie Bourguignon, Claudia Sudol, Damien Brégeon, Djemel Hamdane, Yuri Motorin, Mark Helm

**Affiliations:** Institute of Pharmaceutical and Biomedical Sciences, Staudingerweg 5, Johannes Gutenberg University Mainz, 55128 Mainz, Germany; Institute of Pharmaceutical and Biomedical Sciences, Staudingerweg 5, Johannes Gutenberg University Mainz, 55128 Mainz, Germany; Université de Lorraine, CNRS, INSERM, UAR2008/US40 IBSLor, EpiRNA-Seq Core Facility, 9 Av. De la Forêt de Haye, 54500 Vandoeuvre-lès-Nancy, France; Université de Lorraine, CNRS, UMR7365 IMoPA, 9 Av. De la Forêtde Haye, 54500 Vandoeuvre-lès-Nancy, France; Université de Lorraine, CNRS, INSERM, UAR2008/US40 IBSLor, EpiRNA-Seq Core Facility, 9 Av. De la Forêt de Haye, 54500 Vandoeuvre-lès-Nancy, France; Université de Lorraine, CNRS, UMR7365 IMoPA, 9 Av. De la Forêtde Haye, 54500 Vandoeuvre-lès-Nancy, France; Sorbonne University, CNRS, Institute of Biology Paris Seine, Biology of Aging and Adaptation, 7 quai Saint Bernard, 75252 Paris, France; Collège de France, Sorbonne Université, CNRS, Laboratoire de Chimie des Processus Biologiques (LCPB), 11place Marcelin Berthelot, 75231 Paris France; Sorbonne University, CNRS, Institute of Biology Paris Seine, Biology of Aging and Adaptation, 7 quai Saint Bernard, 75252 Paris, France; Collège de France, Sorbonne Université, CNRS, Laboratoire de Chimie des Processus Biologiques (LCPB), 11place Marcelin Berthelot, 75231 Paris France; Université de Lorraine, CNRS, INSERM, UAR2008/US40 IBSLor, EpiRNA-Seq Core Facility, 9 Av. De la Forêt de Haye, 54500 Vandoeuvre-lès-Nancy, France; Université de Lorraine, CNRS, UMR7365 IMoPA, 9 Av. De la Forêtde Haye, 54500 Vandoeuvre-lès-Nancy, France; Institute of Pharmaceutical and Biomedical Sciences, Staudingerweg 5, Johannes Gutenberg University Mainz, 55128 Mainz, Germany

## Abstract

Various transfer RNA (tRNA) modifications have recently been shown to regulate stress-dependent gene expression by modulating messenger RNA translation. Among these modifications, dihydrouridine stands out for its increase of tRNA structural flexibility. However, whether and how dihydrouridine synthesis reacts to environmental stimuli is largely unknown. In this study, we manipulated the intracellular redox state of *Escherichia coli* using paraquat, revealing differential sensitivities of the three tRNA-dihydrouridine synthases towards oxidative stress. Using liquid chromatography–mass spectrometry quantification of dihydrouridine in various knockout strains, we validated the use of a specific RNA sequencing method, namely AlkAnilineSeq, for the precise mapping of dihydrouridines throughout *E. coli* tRNAs. We found DusA showing high activity, followed by DusB and DusC, whose activity was decreased under paraquat treatment. The relative sensitivity is most plausibly explained by a paraquat-dependent drop of NADPH availability. These findings are substantiated by *in vitro* kinetics, revealing DusA as the most active enzyme, followed by DusB, while DusC showed little activity, likely related to the efficacy of the redox reaction of the flavin coenzyme with NADPH. Overall, our study underscores the intricate interplay between redox dynamics and tRNA modification processes, revealing a new facet of the regulatory mechanisms influencing cellular responses to oxidative stress.

## Graphical Abstract

**Graphical Abstract:**
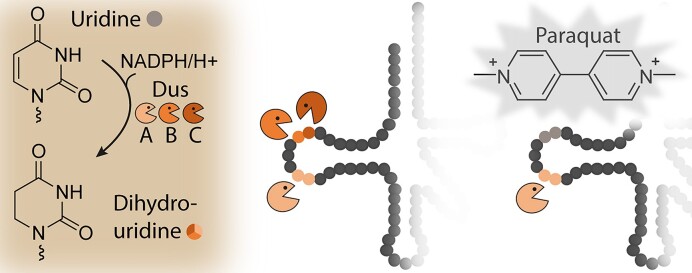

## Introduction

Post-transcriptional modifications enable RNA to fulfil the high variety of roles within the cell for which the canonical nucleosides alone are not sufficient. Transfer RNAs (tRNAs) as one of the oldest RNA species have undergone extensive functional optimization in the course of evolution, thereby amassing the highest density of modifications yet known in RNA. Those tRNA modifications directly involved in translation and decoding fidelity occur in the anticodon loop, while modifications that contribute to RNA stability and folding are typically found in the structural core of the three-dimensional L-shape (1–3). One of the latter modifications is dihydrouridine (D), typically found in tRNA, with an exceptional occurrence reported in *Escherichia coli* 23S ribosomal RNA (4,5). Recent new sequencing approaches lead to detection of further D modifications and to reports on the discovery of modification sites in eukaryotic messenger RNA (mRNA) (6–8).

The reduction of the 5,6-double bond of uridine results in the only non-aromatic nucleobase structure thus far described in RNA (9). While other modifications may lead to a preferential C3′-*endo* pucker of the ribose, dihydrouridine’s saturated ring shows the twist half-chair conformation that leads to a preferred C2′-*endo* sugar pucker (10–14) associated with increased flexibility of the RNA (15). As an example of how D impacts RNA structure, it was shown to trigger loop formation in specific oligonucleotides (16,17). Of note, Nomura *et al.* reported that a lack of dihydrouridine at position 20a in tRNA^Ser^ led to a decrease of the melting temperature indicating that D can contribute to RNA stability even though D was shown to also increase the structural flexibility of tRNA (18).

Dihydrouridylation is catalysed by dihydrouridine synthases, flavin-containing enzymes (19). The prosthetic flavin group is reduced by a redox equivalent NADPH and subsequently transfers the electrons in the form of a hydride to the uridine. The oxidized flavin can be regenerated by another equivalent NADPH, initiating a new catalytic cycle (20). Rider *et al.* reported that the Dus2 enzyme in *Saccharomyces cerevisiae* requires other tRNA modifications, suggesting that the dihydrouridine formation is a late step in tRNA maturation. Furthermore, their findings highlighted the crucial role of a conserved catalytic cysteine, serving as an acid residue essential for the protonation of the enolate intermediate subsequent to the hydride transfer step from the flavin to the RNA substrate (19,21,22). Three different dihydrouridine synthases known in *E. coli* site-specifically modify uridines in the tRNA D-loop (23,24). Reportedly, DusA reduces positions 20 and 20a, while DusB is specific for position 17 and DusC for position 16 in the D-loop (23,25,26). The investigation of dihydrouridine and the catalysing enzymes is also important in a biomedical context. For example, increased levels of eukaryotic dihydrouridine synthases were reported in lung tumours (27) and human Dus2 is involved in the regulation of the interferon-induced protein kinase (28).

Liquid chromatography coupled to mass spectrometry (LC–MS) is the method of choice for structural elucidation as well as quantification of modified RNA residues (29–31). However, analysis at the nucleoside level does not provide information on the modified positions, in contrast to different methods based on next-generation sequencing (NGS) technology. Recently, two NGS-based approaches for the detection of dihydrouridine have been reported. By treating the RNA with sodium borohydride, Draycott *et al.* were able to detect D sites in several RNAs, including mRNA. Borohydride treatment leads to the reduction of D to tetrahydrouridine, removing a hydrogen bond donor on the Watson–Crick face and resulting in RT (reverse transcription) stops (6). Finet *et al.* developed the rhodamine sequencing approach, where RNA is labelled with rhodamine, resulting in RT stops at the respective D sites (7,32). A mutant of the respective D-synthase is required to distinguish between Dus-dependent and Dus-independent RT stops. Draycott *et al.* reported that the D-Seq has specific blind spots, as it is the case for D at position 47, because this site is too close to the 3′ end of the tRNA transcript. However, the advantage of both methods is that they are suitable for any type of RNA. Beyond these, we here expand on the previous report that D modifications can be detected site-specifically based on a hitherto unexploited molecular mechanism. The corresponding sequencing approach, called AlkAnilineSeq (AAS), was originally developed to detect m^7^G and m^3^C. AAS is based on the formation of an abasic site as a result of alkaline conditions, to which aniline can then be added, leading to a β-elimination, resulting in strand breaks detectable by sequencing. As the non-aromatic ring of dihydrouridine undergoes ring opening under mild alkaline conditions (33,34), AAS is able to detect D sites. Previously, signals were observed at positions known to be dihydrouridylated, but further method validation for the detection of D had not been conducted (35,36).

**Figure 1.**
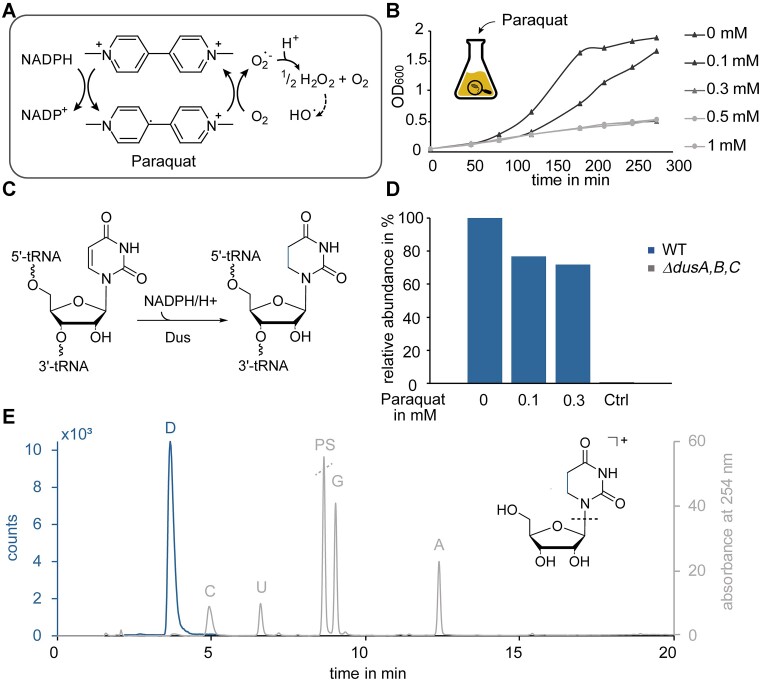
Effect of paraquat treatment on bacterial growth and dihydrouridine modification levels in *E. coli*. (**A**) Redox cycle of paraquat with oxygen. The scheme is adapted from Gray *et al.* (45). (**B**) Growth behaviour of *E. coli* cultures after paraquat treatment with different concentrations. (**C**) Reduction of uridine to dihydrouridine catalysed by the dihydrouridine synthase (Dus) requiring NADPH as redox equivalent. (**D**) LC–MS/MS analysis of dihydrouridine levels in total tRNA isolated from wild-type (WT, blue) *E. coli* cultures treated with paraquat (0, 0.1 and 0.3 mM). tRNA isolated from a knockout strain of all three dihydrouridine synthases (*ΔdusA,B*,*C*) served as a negative control. The signals were normalized to the respective ultraviolet (UV) signal of adenosine and related to the signal of tRNA isolated from untreated WT culture. (**E**) Chromatographic separation of dihydrouridine (D, blue) in comparison to the main nucleosides cytidine (C), uridine (U), guanosine (G) and adenosine (A) (grey) on an RP C18 HPLC column. Dihydrouridine was detected by mass spectrometry, while the signals of the main nucleosides and the signal of the deaminase inhibitor pentostatin (PS) were detected by UV spectrometry at a wavelength of 254 nm.

Disturbances of the sensitive redox balance in the cell can cause severe defects in the biological system. The redox-active and therefore toxic bipyridine salt paraquat can be used to analyse effects related to intracellular redox states, as it was shown to deplete cellular levels of NADPH (37). The latter plays an essential role in the redox homeostasis, as the dinucleotide is cofactor of the glutathione and thioredoxin reductases, which in turn are involved in the antioxidant activity by scavenging hydrogen peroxides (38). With its bipyridine structure, paraquat easily undergoes one-electron reduction, leading to a mesomerically stabilized radical, delocalized in the ring structure. This radical can in turn be involved in a redox cycle with oxygen leading to a reoxidation of the paraquat radical and the reduction of oxygen forming a superoxide anion radical (O$_{2}^{\bullet-}$). The latter can react further to hydrogen peroxide and oxygen catalysed by the superoxide dismutase, with hydrogen peroxide further dissociating into hydroxyl radicals (HO$^{\bullet}$) or leading to the formation of other reactive oxygen species catalysed by the superoxide dismutase (Figure 1A) (39–45). This so-called redox cycling can be induced by NAD(P)H oxidases generating the paraquat radical as, for example, the nitric oxide synthase or cytochrome P450 (46,47). Analysing the effect of paraquat in two different *E. coli* strains, Kitzler *et al.* observed that the *E. coli* strain K-12 appeared to be resistant to the lethal effect of paraquat in contrast to the *E. coli* strain B. They found out that the latter strain retains paraquat for many hours, while paraquat can diffuse rapidly through the K-12 cells increasing their viability (48). Treating *E. coli* K-12 with paraquat, Leiva *et al.* reported the inactivation of tRNA^Gly^ as well as a much slower translation initiation, thus modulating the translation under oxidative stress (49,50).

In this study, we investigate the redox dependency of tRNA dihydrouridylation in *E. coli* by supplementing the bacterial cultures with paraquat to induce redox changes. The growth under aerobic and anaerobic conditions allowed the analysis of oxygen dependency and resulted in defined growth conditions to ensure reproducibility. Polysome preparations enabled the analysis of dihydrouridine’s role during translation and the culturing of Dus knockout strains revealed that the enzymes were differentially affected by redox changes. *In vitro* assays of the Dus enzymes indicated that these observations might be the result of differential enzyme activities and substrate specificities. This comprehensive approach provides valuable insights into the intricate relationship between redox dynamics, tRNA modification and translation processes in *E. coli*.

## Materials and methods

### Growth of *E. coli* strains and supplementation with paraquat

*Escherichia coli* strains cultured in flasks were grown in LB medium (20 g/l, LB Broth Lennox, Sigma–Aldrich) at 37°C and 190 rpm. Paraquat referred to as paraquat dichloride hydrate (Sigma–Aldrich) was supplemented in the indicated concentrations at the beginning of cultivation (growth curves) or at an optical density (OD_600_) of 0.4 for 3 h.

Under defined aerobic or anaerobic conditions, the cultures were grown in a Duran glass reactor in combination with a magnetic stirrer (300 rpm). Constant temperature was ensured using a water bath at 37°C. The strains were grown in 0.5× LB medium (10 g/l, LB Broth Lennox, Sigma–Aldrich) and paraquat was supplemented at an optical density (OD_600_) of 0.1. Afterwards, the culture was continued until the bacteria reached an optical density of 0.3 or 0.6 in case of polysome preparations.

The *E. coli* wild-type strain (Keio parent, BW25113) and the Dus single knockouts (*ΔdusA*, *ΔdusB* and *ΔdusC*) were purchased from the *E. coli* Keio knockout collection [GE Healthcare (Dharmacon™), UK]. The triple knockout *(ΔdusA,B*,*C*) was produced as described in (51).

### Extraction of total tRNA from *E. coli* cultures

After cell harvesting (10 000 × *g*, 4°C, 10 min), the cell pellets were resuspended in TRI Reagent (Sigma–Aldrich) and the total tRNA was isolated following the instructions of the manufacturer. The dried RNA pellets were dissolved in Milli-Q water.

### *Escherichia coli* polysome preparations

For polysome preparations, chloramphenicol (100 μg/ml, Carl Roth) was added to the *E. coli* cultures when the optical density of 0.6 was reached. After an incubation for further 3 min, the cells were harvested (10 000 × *g*, 4°C, 10 min) and the cell pellets were resuspended in lysis buffer (100 mM NH_4_Cl, 10 mM MgCl_2_, 20 mM Tris, pH 7.5). Lysozyme (Carl Roth) was added, the bacteria suspension was subjected to freeze–thaw cycles in liquid nitrogen and finally 10% deoxycholate (Sigma–Aldrich) was supplemented. After a centrifugation step (12 000 × *g*, 4°C, 10 min) to separate remaining cell wall debris, the supernatant was loaded on top of a sucrose gradient (5–40%) that was generated using a Biocomp gradient station model 108 (settings: time 1.24 min, angle 81.5°, speed 21 rpm). The cell lysate was separated along the sucrose gradient by ultracentrifugation [Beckman Ultracentrifuge Optima LE-80K, SW40 Ti rotor (Beckman Coulter)] at 150 000 × *g* and 4°C for 2.5 h. Subsequently, the gradients were fractionated in four fractions (F0, F1, F2 and F3) by measuring the absorbance at 260 nm (Biocomp Gradient Station model 108 combined with Gilson Fraction Collector FC203B). To isolate the total RNA from the collected fractions, TRI Reagent (Sigma–Aldrich) was added and the extraction was performed as described in the manufacturer’s protocol.

### Isolation of tRNA from *E. coli* polysome preparations

The total RNA isolated from the polysome preparations was further separated using gel electrophoresis [10% denaturing polyacrylamide gel electrophoresis (PAGE)]. After staining with GelRed (Biotium) and visualizing using a Typhoon 9400 (excitation wavelength 532 nm), the corresponding tRNA bands were excised from the gel and eluted in 0.5 M ammonium acetate overnight. Remaining gel pieces were removed using NanoSep filters and the tRNA eluate was precipitated using ethanol. After washing with 70% ethanol, the tRNA pellets were dried and subsequently resuspended in Milli-Q water.

### Quantification of dihydrouridine levels by LC–MS/MS

To analyse the samples by LC–MS/MS, the tRNA was digested to nucleoside level using 0.6 U nuclease P1 from *Penicillium citrinum* (Sigma–Aldrich), 0.2 U snake venom phosphodiesterase from *Crotalus adamanteus* (Worthington), 2 U FastAP (Thermo Scientific), 10 U benzonase (Sigma–Aldrich) and 200 ng pentostatin (Sigma–Aldrich) in 25 mM ammonium acetate buffer at pH 7.5 (Sigma–Aldrich) overnight at 37°C.

Three hundred nanograms of total tRNA or 100 ng of tRNA isolated from polysomal fractions was separated on a Synergi Fusion RP18 column (4 μm particle size, 80 Å pore size, 250 mm × 2.0 mm; Phenomenex) using an Agilent 1260 LC series. Ammonium acetate buffer (5 mM, pH 5.3) was used as solvent A and was combined with acetonitrile as solvent B (LC–MS grade, Honeywell). The gradient started with 100% solvent A (flow rate 0.35 ml/min) and was followed by a linear gradient to 8% solvent B at 10 min. Subsequently, the gradient was raised to 40% of solvent B after 20 min and restored afterwards for 3 min to the initial conditions, which were held for further 10 min. A diode array detector recorded the UV signal (254 nm) monitoring the eluting main nucleosides. After chromatographic separation, the MS/MS analysis of the first experiment was performed using an Agilent 6460 Triple Quadrupole mass spectrometer equipped with an electrospray ion source (ESI; parameters: gas temperature 350°C, gas flow 8 l/min, nebulizer pressure 50 psi, sheath gas temperature 350°C, sheath gas flow 12 l/min, capillary voltage 3000 V and nozzle voltage 500 V). Further analysis was performed using an Agilent 6470 Triple Quadrupole mass spectrometer with the following ESI parameters: gas temperature 300°C, gas flow 7 l/min, nebulizer pressure 60 psi, sheath gas temperature 400°C, sheath gas flow 12 l/min and capillary voltage 3000 V. The mass spectrometer was run in positive ion mode using the dynamic multiple reaction monitoring mode searching for the mass transition of dihydrouridine (*m/z* 247 → 115) (Agilent Mass Hunter software). Relative quantification was performed as described in (30). Briefly, the area under the curve of the detected dihydrouridine signals was normalized to the UV signal of adenosine, which has proven to be the most suitable as it provides stable UV signals that do not overlay with other UV signals. Guanosine co-elutes with the pentostatin added during sample preparation, which might falsify the UV signal intensity. Uridine and cytosine are considered less suitable as they elute rather early and the signals may overlay with UV signals of DNA in case of contaminations.

LC–MS data are available as Supplementary Table S1.

### Analysis of NADPH levels in *E. coli* by LC–MS/MS

To analyse the NADPH levels as a result of the presence of paraquat, *E. coli* wild-type cultures were grown under defined aerobic conditions, supplemented with 0.3 mM paraquat at an optical density of 0.1 and harvested at an optical density of 0.6. Metabolites were extracted according to the extraction protocol of Thorfinnsdottir *et al.* (52). Briefly, bacterial cultures were filtered through hydrophilic filters (0.45 μm, Merck Millipore) and the retained metabolites were subsequently eluted from these filters in a cold extraction step in methanol, water and acetonitrile (2:5:3, v/v%) at −15°C, followed by centrifugation (4500 × *g*, −9°C, 10 min) and lyophilization of the supernatant. The lyophilized metabolites were resuspended in water and filtered through 10 kDa cut-off filters (Amicon, Merck Millipore). The flow-through was incubated on 0.1 g POLYGOPREP 60–50 C18 silica gel (Macherey-Nagel) before being filtered through 3 kDa cut-off filters (Vivaspin, Merck) and subjected to LC–MS/MS measurement.

The levels of NADPH were analysed using an Agilent 1260 LC series (Poroshell 120EC-C18, 3.0 mm × 150 mm, 2.7 μm, Agilent) in combination with an Agilent 6470 Triple Quadrupole mass spectrometer (ESI parameters: gas temperature 350°C, gas flow 8 l/min, nebulizer pressure 50 psi, sheath gas temperature 350°C, sheath gas flow 12 l/min, capillary voltage 3000 V and nozzle voltage 500 V). Solvent A was 20 mM ammonium acetate (pH 6), which was combined with acetonitrile (solvent B, LC–MS grade, Honeywell). The gradient started in the first minute with 100% solvent A (flow rate 0.35 ml/min). At the beginning, the percentage of solvent B was slowly increased up to 30% within 9 min, followed by a steeper increase up to 90% solvent B within another 5 min. The latter was maintained for 9 min until the initial conditions are restored after 14 min, which were held for a further 8 min. The mass spectrometer was operated in positive ion mode using the multiple reaction monitoring mode searching for the mass transition of NADPH (746 → 729). An external calibration curve of NADPH standard solutions (Sigma–Aldrich) was used to quantify the NADPH levels in the analysed extracts.

### Detection of dihydrouridine by AAS

About 100 ng of gel purified tRNAs was subjected to AAS (35,36). Briefly, tRNA was subjected to fragmentation by a mild alkaline hydrolysis for 5 min at 96°C. D rings are unstable under these conditions and were cleaved. Fragments generated were end-repaired by extensive treatment with alkaline phosphatase to remove both pre-existing 5′-P and 3′-P resulting from alkaline hydrolysis. tRNA fragments were then subjected to aniline treatment, resulting in deprotection of a 5′-phosphate at the *N* + 1 nucleotide, which serves as competent 5′-phosphate for selective ligation of sequencing adapters. Libraries were prepared using the NEBNext^®^ Small RNA Library Prep Set for Illumina^®^ using the manufacturer’s recommendations. Libraries were then qualified, quantified and multiplexed for high-throughput sequencing using a NextSeq2000 with a 50-bp single-end read mode.

Initial trimming of adapter sequence was done using Trimmomatic-0.32 (53) with the default parameters. Alignment to the reference tRNA sequence was done by Bowtie2 (ver 2.4.4) in end-to-end mode and with ‘sensitive parameter’ set. Counting of the mapped reads and positions of their 5′-extremities was performed using awk command (54). Coverage for reference sequence was calculated using samtools mpileup command. 5′-End count was directly used for calculation of AAS scores. Stop ratio (ratio of reads starting at a given position and total number of passing reads) for every position of the reference sequence was calculated using 5′-end count and coverage data. All other steps of analysis were performed in R Studio 1.0.143 with R version 3.4.4.

### *In vitro* kinetic activity assay of the *E. coli* dihydrouridine synthases

The ability of *E. coli* Dus to oxidize NADH and NADPH under steady-state conditions was determined in the presence of air, as final electron acceptor, in 50 mM HEPES (pH 7.5), 150 mM NaCl and 15% glycerol (v/v). Assays were performed using various concentrations of NAD(P)H. The amount of NAD(P)H oxidized was monitored by following the decrease of absorbance at 343 nm (*ϵ*_343_ = 6.21 mM^−1^ cm^−1^). The initial rate versus NAD(P)H concentration was analysed according to the Michaelis–Menten formalism. The *in vitro* kinetic dihydrouridylation assay of *E. coli* dihydrouridine synthases involved assessments at various time points (0 min, 1 min, 5 min, 15 min, 20 min and 1 h) at 37°C. The reaction took place in a buffer comprising 100 mM Tris–HCl (pH 8), 150 mM ammonium acetate, 2 mM dithiothreitol, 10 mM MgCl_2_ and 250 μM FMN (Flavin mononucleotide). Bulk tRNAs (20 μM), derived from the triple mutant strain, were incubated with 5 μM of protein in a total volume of 100 μl. The reaction commenced upon the addition of 2 mM NADPH. Quenching was achieved by adding 100 μl of acidic phenol (Sigma–Aldrich), followed by a 10-min centrifugation at 16 000 × *g*. tRNAs in the aqueous phase were ethanol precipitated and further purified using a MicroSpin G-25 column (GE Healthcare). Dihydrouridine quantification was conducted through LC–MS spectrometry analysis.

## Results

### Effect of paraquat treatment on dihydrouridine levels in *E. coli*

Because of its toxicity, the impact of paraquat on the bacterial growth was investigated supplementing *E. coli* cultures in a pilot experiment, conventionally grown in conical flasks in an incubator shaker, with paraquat at different concentrations. With the aim to identify conditions that impaired growth without killing bacteria in significant numbers, we first tested a concentration range. Supplementation of 0.1 mM paraquat resulted in moderate deceleration of the growth, while higher concentrations (0.3, 0.5 and 1 mM) caused severe reduction of the growth rate (Figure 1B). To explore a range of growth speeds in a pilot characterization, we selected 0.1 and 0.3 mM, since higher concentrations did not show any difference except potentially creating elevated levels of dead cells that might skew the results. As paraquat is known to change the redox equilibrium in the cell and the reduction of uridine to dihydrouridine in turn depends on the redox equivalent NADPH (Figure 1C), it was furthermore analysed whether paraquat affected the dihydrouridine modification levels in *E. coli* tRNA. Total tRNA isolated from wild-type *E. coli* cultures treated with 0.1 and 0.3 mM paraquat was subjected to LC–MS/MS analysis (Figure 1D). The dihydrouridine modification changes the hydrophobicity of the nucleobase and leads to altered chromatographic properties relative to uridine (U). On a reverse phase C18 HPLC column, D elutes earlier in comparison to U as depicted in Figure 1E. After chromatographic separation, D was quantified via mass spectrometry, based on the common fragmentation of ­132 Da corresponding to the loss of ribose. As shown in Figure 1D, the modification levels decreased in cultures that were treated with paraquat by 23% and 28%, respectively. Culturing a triple knockout strain (*ΔdusA,B,C*, grey) served as a negative control; here, only a marginal dihydrouridine level was detected (below 1%).

Following up on the known redox cycling of paraquat by ambient oxygen, we attempted to measure and control the latter. As this turned out to be technically challenging, we concluded from the pilot experiment a need for controlled growth conditions and continuous monitoring of the oxygen saturation in the growth medium in a different setting.

### Oxygen-dependent effect of paraquat on dihydrouridine levels in *E. coli* tRNA

**Figure 2.**
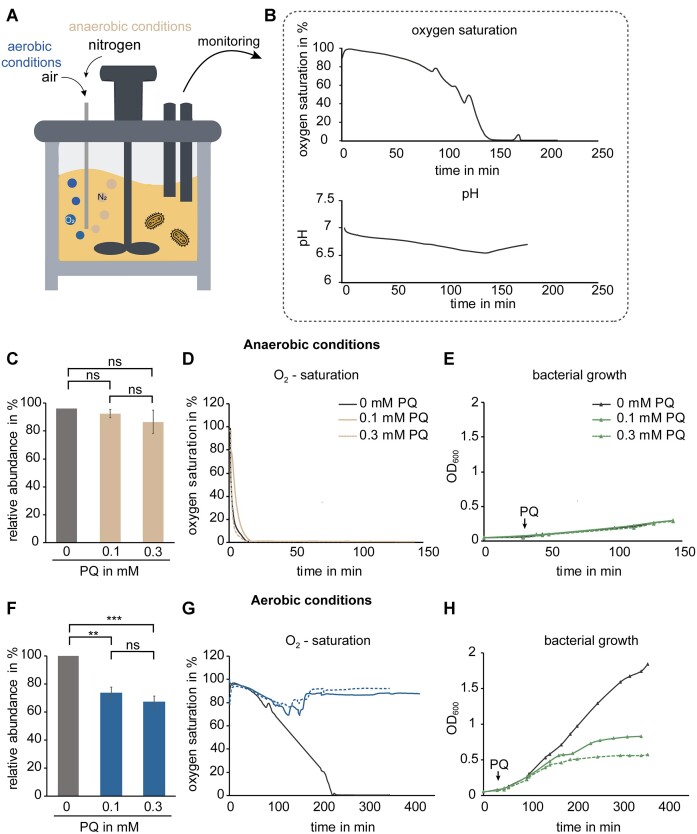
Defined conditions of bacterial growth in the presence of paraquat and LC–MS/MS analysis of dihydrouridine modification levels in tRNA isolated from these cultures. (**A**) Cultivation of bacteria in a bioreactor with a constant air or nitrogen stream to generate controlled growth conditions. (**B**) Oxygen and pH electrodes enable the continuous monitoring of oxygen saturation and pH value of the medium. (**C**) Levels of dihydrouridine modifications in total tRNA isolated from anaerobic cultivated wild-type *E. coli* cultures untreated (grey) and treated with paraquat (0.1 and 0.3 mM, beige) determined by LC–MS/MS. The signals were normalized to the respective UV signals of adenosine and related to the dihydrouridine signal of tRNA isolated from untreated culture. Results are shown as average of biological triplicates; the error bars depict the standard deviations. Significances were determined using Student’s *t*-test (ns: not significant). (**D**) Monitoring of oxygen saturation of treated (beige) and non-treated (grey) cultures. (**E**) Bacterial growth behaviour of treated (green) and non-treated (grey) cultures. The addition of paraquat (PQ) at an optical density of 0.1 is marked by an arrow. (**F**) Dihydrouridine levels in tRNA from *E. coli* cultures grown under aerobic conditions. Results are shown as average of biological triplicates of untreated (grey) and treated (blue) cultures. Normalization and statistical analysis were performed similarly to C (ns: not significant, **P < 0.01, ***P < 0.001). (**G**) Oxygen saturation of bacterial cultures treated (blue) or non-treated (grey) with paraquat (0.1 mM: solid line; 0.3 mM: dashed line). (**H**) Monitoring of the optical density of bacterial cultures treated (green) and non-treated (grey) with paraquat (0.1 mM: solid line; 0.3 mM: dashed line). The addition of paraquat (PQ) is marked with an arrow.

Based on the redox cycle of paraquat with oxygen described earlier, the experimental set-up was adapted to grow cultures under defined conditions in a bioreactor as shown schematically in Figure 2A. A continuous stream of gas, either air to ensure a constant oxygen supply during aerobic conditions or nitrogen to grow the bacteria under anaerobic conditions, was conducted through the culture medium. Sensors attached to the bioreactor enabled the continuous measurement of the oxygen saturation and the pH value as shown exemplarily for a culture under aerobic conditions (Figure 2B).

The D levels in tRNA isolated from *E. coli* cultures, anaerobically grown in an N_2_ gas stream, were determined by LC–MS/MS. Comparing the signals of tRNA isolated from untreated (grey) and treated cultures (beige), no significant difference as a result of the paraquat treatment was detectable (Figure 2C). As expected, under anaerobic conditions, oxygen saturation strongly decreased immediately after the start of cultivation (Figure 2D). Interestingly, the bacteria showed continued albeit slow growth even when the measured oxygen saturation of their growth medium had decreased to 0%. Of note, no effect of the paraquat treatment on the bacterial growth was observable under anaerobic conditions (Figure 2E).

In contrast, under aerobic conditions a significant decrease of D levels was detectable when the cultures were treated with paraquat. tRNA isolated from the culture supplemented with 0.1 mM paraquat showed a decrease of 27% as average of three biological replicates, while the 0.3 mM culture resulted in a decrease of 33% in comparison to the non-treated one (grey). Direct comparison of the two different paraquat concentrations showed small differences that were not statistically significant (Figure 2F). At this point, we exemplarily confirmed the assumed paraquat-induced depletion of NADPH levels by LC–MS. The data in Supplementary Figure S1 reveal a strong drop of at least 3-fold in cultures exposed to 0.3 mM paraquat.

Contrary to the growth behaviour under anaerobic conditions, the bacterial growth was slowed down when paraquat was supplemented (green) under aerobic conditions, similar to the pilot experiments (Figure 1B). The oxygen saturation of the non-treated culture (dark grey line) decreased slowly and in correspondence to the bacterial growth. Interestingly, the treated cultures (blue) showed an increase of the oxygen saturation instead of the decrease observed for the non-treated culture (Figure 2G and H). The decreased oxygen consumption can presumably be attributed to the decelerated growth rate, induced by paraquat.

We conclude that paraquat affects the dihydrouridylation of *E. coli* tRNAs only in the presence of oxygen and not under anaerobic conditions, in keeping with the functional connection known from the redox cycling of paraquat.

### Different activities of dihydrouridine synthases

**Figure 3.**
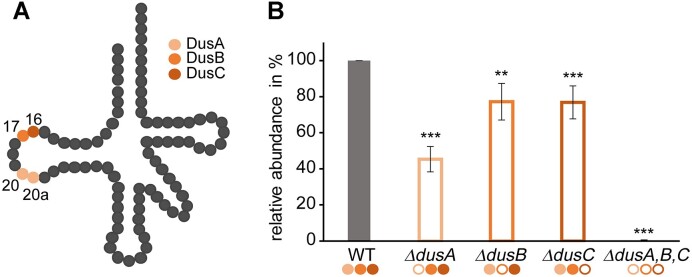
Detection of dihydrouridine modification levels by LC–MS/MS in tRNA isolated from *E. coli* knockout strains. (**A**) Schematic tRNA highlighting the uridine positions modified site-specifically by the three different dihydrouridine synthases DusA (salmon), DusB (orange) and DusC (red). (**B**) LC–MS/MS analysis of dihydrouridine levels in tRNA isolated from bacterial cell lysates of single knockout strains of the three Dus enzymes. The signals were normalized to the UV signal of adenosine and related to the wild-type signal. Experiments were performed in biological replicates (*n* = 5) and results are shown as average; the standard deviations are depicted as error bars. A two-tailed Student’s *t*-test was used to determine significances between wild-type and each knockout (***P < 0.001, **P < 0.01). Filled circles mark active enzymes, while empty circles indicate the absence of the enzyme.

In order to analyse the respective percentage of D modifications specifically introduced by the respective dihydrouridine synthases (Figure 3A), tRNA isolated from single knockout strains was analysed by LC–MS/MS to determine the D levels in strains missing one of the Dus enzymes. tRNA isolated from *ΔdusA* contained 55% less D than tRNA isolated from the wild-type strain. tRNA from the other single knockout strains showed a reduction of 23% in *ΔdusB* and 23% in *ΔdusC*. tRNA from the knockout strain lacking of all three Dus enzymes showed negligible signals, effectively serving as a negative control (Figure 3B).

These results indicate that the three different Dus enzymes modify non-overlapping sets of D residues and the reduction of D levels in the single knockouts lets us conclude that DusA introduces about half of all dihydrouridines, and in line with a previous report from Bishop *et al.* (25), DusB and DusC account for biosynthesis of the other half, although the numbers above leave room for potential redundancies, i.e. sites that might be modified by more than one Dus enzyme.

### Detection of dihydrouridine modifications by AAS reveals position-specific impact of paraquat

**Figure 4.**
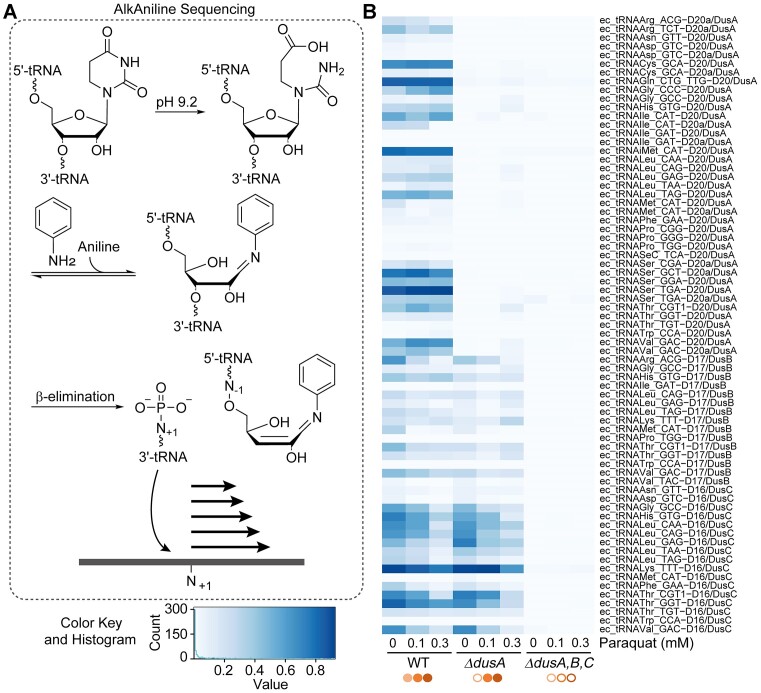
Detection of dihydrouridine modification sites by AAS. (**A**) Schematic illustration of the AAS. (**B**) Analysis of total tRNA isolated from treated (0.1 and 0.3 mM paraquat) and untreated *E. coli* wild-type and knockout cultures by AAS. The different tRNAs and respective positions of dihydrouridine modifications are indicated on the right. Results are shown as stop ratio (ranging from 0 to 1). Filled circles mark the active Dus enzymes, while empty circles indicate the knocked-out enzyme.

In addition to LC–MS/MS analysis, we further developed AAS into a site-specific detection of D (Figure 4A). Prior experiments with tRNA from various organisms had consistently yielded signals at sites known to contain D, but a more comprehensive validation was missing. As a first assessment of the method, tRNA samples from the pilot experiment were analysed, in which LC–MS/MS analysis had revealed a decrease of dihydrouridine modifications as a result of the paraquat treatment (Figure 1D). In terms of method validation, it was expected that the decreased D content under paraquat treatment, as evidenced by LC–MS/MS, would also be reflected in the AAS results, thereby including some position-specific information. We furthermore reasoned that analysis by AAS of cultures of a single knockout strain of DusA (*ΔdusA*) and the triple knockout strain (*ΔdusA,B,C*), treated with 0.1 and 0.3 mM paraquat, would provide additional context to assess the performance of AAS. This was indeed the case, as shown in Figure 4.

In tRNA isolated from the wild-type strain, signals were detected at respective dihydrouridine positions 16, 17, 20 and 20a, while, apart from some noise, tRNA isolated from *ΔdusA* only showed signals at positions 16 and 17 corroborating the position specificity of DusA for positions 20 and 20a.

In the AAS scoring system termed ‘stop ratio’ (see the ‘Materials and methods’ section), scaled from 0 to 1, all signals of the triple knockout *ΔdusA,B,C* were consistently below 0.04. Concerning the paraquat treatment, interestingly, most of the detected D signals at positions 20 and 20a did not show a decrease as a result of the paraquat treatment, while D signals at positions 16 and 17 did decrease (Figure 4B). Of note, while all DusC-related signals at position 16 clearly decreased upon paraquat stress, this was only detectable for around 56% of the DusB-related signals at position 17.

We conclude that the results detected by AAS are in agreement with the LC–MS/MS results, thus, in combination with results obtained from the knockout mutants, validating AAS for sequence-specific D detection. Further, these results led us to conclude that the DusA enzyme is markedly less sensitive towards redox changes introduced by paraquat compared to DusB and DusC, and that, among the latter, DusC is more sensitive than DusB.

### Dihydrouridine levels of polysome-bound tRNAs

**Figure 5.**
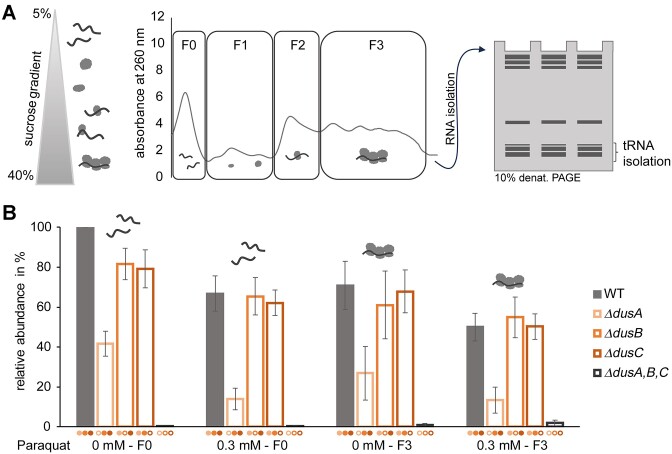
Polysome preparations of *E. coli* cultures treated with paraquat and analysis of isolated tRNA. (**A**) Schematic workflow of polysome preparation. Fractionation of *E. coli* cell lysate on a sucrose gradient (5–40%) after ultracentrifugation; different fractions were detected by absorbance at 260 nm. Subsequently, total RNA and then tRNA were isolated from these fractions. (**B**) LC–MS/MS analysis of dihydrouridine in tRNA isolated from free tRNA fraction (F0) and polysomal fraction (F3) of treated (0.3 mM paraquat) and untreated wild-type (grey) and knockout cultures (*ΔdusA*: salmon; *ΔdusB*: orange; *ΔdusC*: red). Filled circles indicate active enzymes and empty circles mark the knocked-out enzyme. LC–MS/MS signals were normalized to the UV signal of adenosine and the normalized signal of D in tRNA isolated from free RNA fraction of untreated wild-type culture (WT 0 mM F0) was set to 100%. Experiments were performed in biological triplicates and results are shown as average; the standard deviations are depicted as error bars. Statistical significances are shown in Supplementary Figure S2.

The structural flexibility that dihydrouridine confers to tRNAs led us to hypothesize that it might facilitate accommodation of the latter on the ribosome. To investigate this hypothesis, we quantified the D content of tRNAs isolated from actively translating polysomes. These tRNAs are, by definition, functional in their interaction with cognate aminoacyl-tRNA synthetases, as well as with EF-Tu. To analyse whether the dihydrouridine modification plays a role in translation and whether this role might be affected by redox state, polysomes of paraquat-treated and non-treated *E. coli* cultures were prepared. The higher paraquat concentration of 0.3 mM was used within these experiments to analyse the bacterial phenotype most likely to display a robust effect by paraquat supplementation. Ultracentrifugation of cell lysate, which was loaded on top of a sucrose gradient, leads to the accumulation of low-density molecules and complexes in the upper part of the gradient, including unbound tRNAs, while more dense particles, here including polysome complexes, are found in the lower part. After fractionation of the gradient, total RNA was extracted and then tRNA was isolated using denaturing PAGE (Figure 5A). The tRNAs isolated from the free RNA fraction in the upper part of the gradient (F0) and tRNAs isolated from polysomal fraction (F3) were digested to nucleoside level and subjected to LC–MS/MS analysis. After normalization to the UV signal of adenosine, the signals were related to the D level of tRNA isolated from the collected fraction F0 of untreated wild type.

In the F0 ‘free tRNA’ fraction, the decrease of D levels in the single knockout strains *ΔdusA*, *ΔdusB* and *ΔdusC* (Figure 5B) was recapitulated as shown before (Figure 3) for the non-polysome preparations. Paraquat treatment showed differential effects on the D levels. Preparations of tRNA from the wild-type strain showed a decrease of 33%, similar to the previous results shown in Figure 2E. D levels in the *ΔdusA* strain were strongly affected by the presence of 0.3 mM paraquat: the D levels decreased by 67% in comparison to the untreated *ΔdusA* culture. In contrast to this, the D levels in *ΔdusB* and *ΔdusC* strains showed only a decrease of 20% and 22%, respectively, as a result of the paraquat supplementation.

On tRNAs that were bound to polysomes in the wild-type strain, lower D levels were detected compared to the D level of the free tRNA pool. However, the D levels on polysomes isolated from *ΔdusA*, *ΔdusB* and *ΔdusC* strains did not show a significant difference to the free tRNA pools in these strains. Paraquat treatment of wild-type and knockout strains did not significantly change the dihydrouridine levels of tRNA from isolated polysomes (Figure 5B; significance values added in Supplementary Figure S2).

We conclude from examination of polysome-bound tRNAs that, under the growth conditions examined, paraquat treatment has no apparent effect. This means that we find no evidence to support our hypothesis that dihydrouridylation might play an accelerating part in the translation process. A detailed positional analysis by AAS, as presented in the next section, supports this conclusion. We further conclude from the LC–MS analysis of the cytosolic pool (F0 fraction) that DusA is less sensitive towards redox stress compared to DusB and DusC as the D levels in tRNA isolated from the DusA knockout strain, which in turn has active DusB and DusC enzymes, were most affected by the paraquat supplementation, since either lower or comparable abundances of D were detected. Again, a positional analysis is presented in the next section.

### AAS confirmed differential Dus activities under paraquat treatment

**Figure 6.**
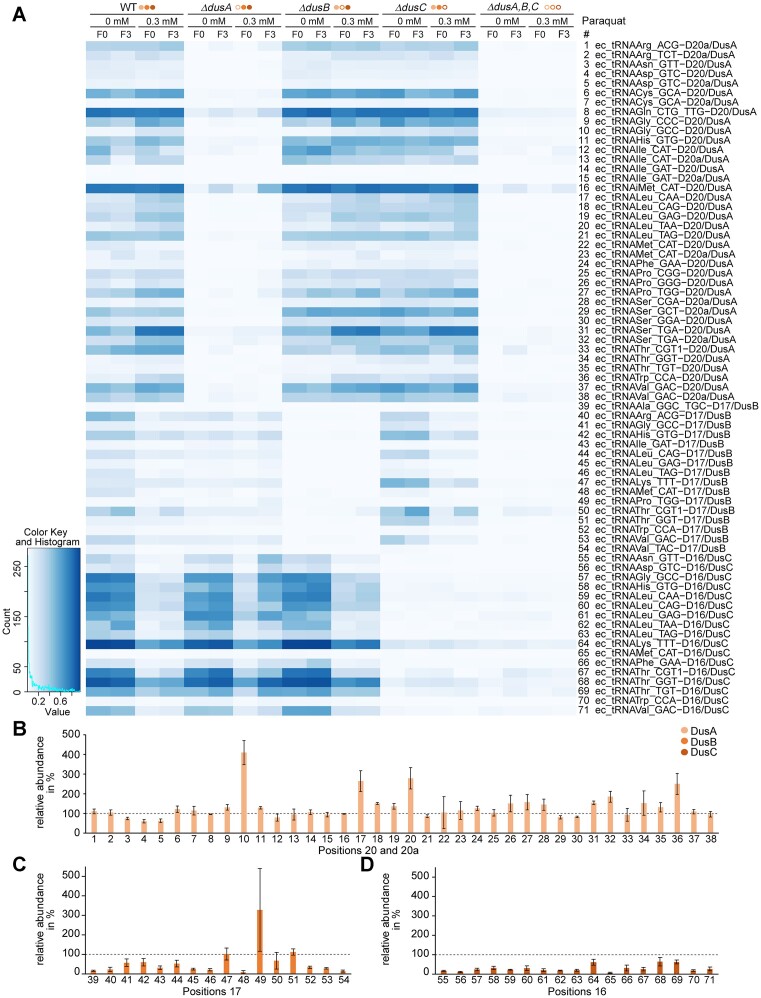
AAS of tRNA isolated from *E. coli* polysome preparations. (**A**) Detection of dihydrouridine modification sites in tRNAs isolated from free RNA fraction (F0) and polysomal fraction (F3) that were obtained from treated (0.3 mM paraquat) and untreated *E. coli* wild-type (WT) and single knockout (*ΔdusA*, *ΔdusB* and *ΔdusC)* cultures. Filled circles mark the active Dus enzymes, while empty circles indicate that the enzyme is knocked out. The different tRNAs and respective positions of dihydrouridine modifications are indicated on the right. Results are shown as stop ratio. Further replicates are provided in Supplementary Data. (**B**) Dihydrouridine signals detected at positions 20 and 20a in tRNA isolated from free RNA fraction of polysome preparations of *E. coli* wild-type cultures treated with 0.3 mM paraquat. Modification sites were detected by AAS and were related to the stop ratio score that was detected in tRNA isolated from untreated culture at the respective tRNA position (set to 100%, dashed line). The tRNAs are numbered according to the tRNAs listed in panel (A). Results are shown as average of three biological replicates; the error bars indicate the standard deviations. (**C**) Dihydrouridine modifications detected at position 17 in tRNA isolated from free RNA fraction of polysome preparations of *E. coli* wild-type cultures treated with 0.3 mM paraquat. Analysis was performed similarly to panel (B) and tRNAs are numbered according to the tRNAs listed in panel (A). (**D**) Detection of dihydrouridine modifications at position 16 in tRNA isolated from free RNA fraction of polysome preparations of *E. coli* wild-type cultures treated with 0.3 mM paraquat. Analysis was performed similarly to panel (B) and tRNAs are numbered according to the tRNAs listed in panel (A).

The tRNA samples isolated from polysome preparations were also analysed by AAS in three biological replicates (Figure 6 and Supplementary Figures S3 and S4). Preparations of tRNA from the respective knockout strains showed predominantly no or low signals at the positions related to the respective knocked-out enzyme, except for a few tRNAs showing signals of moderate intensity. This is in agreement with the LC–MS results and once more recapitulates the position specificity of the enzymes.

Comparison of signals from the free tRNA fraction with the polysomal tRNAs did not reveal any uniform tendency. In some tRNAs, the signal intensity for D residues increased on polysomes, while for others a decrease or no change was detectable. The significant reduction that was observed in the wild-type tRNAs (Figure 5B) cannot be attributed to D levels for specific tRNAs in AAS. We conclude that, at least under the conditions examined, the level of D modification does not improve tRNA recruitment to the active translation machinery.

Interestingly, DusA-specific signals at positions 20 and 20a in wild type, *ΔdusB* and *ΔdusC* were still detected under 0.3 mM paraquat treatment, some even at increased intensity. In contrast, signals detected at positions 16 and 17 mostly decreased under paraquat treatment, confirming the previous assumption that DusA is less sensitive towards redox changes, whereas the activities of DusB and DusC are highly affected. We thus decided to break down paraquat-induced changes to the respective Dus activities by position in more detail.

For an enzyme-specific analysis of the paraquat effect, the sequencing data were normalized to the D signals for tRNAs isolated from free RNA fraction (F0) extracted from the untreated wild-type culture; the latter were thus set to 100% (depicted as dashed line in Figure 6B–D). The signals detected in F0 tRNAs from treated wild-type cultures were related to the respective untreated sample and the results are shown separately for positions 20 and 20a modified by DusA (salmon, Figure 6B), position 17 modified by DusB (orange, Figure 6C) and position 16 modified by DusC (red, Figure 6D) as average of biological triplicates.

DusA-dependent signals detected at positions 20 and 20a were mostly increased or unaffected in the presence of paraquat, apart from a few tRNAs showing a slight decrease (7 tRNAs out of 38). Contrarily, tRNA positions modified by DusB and DusC were highly affected by redox changes induced by paraquat. While a few tRNA positions 17 showed no reduction or, in case of one tRNA, an increase as a result of paraquat, all tRNA positions 16 were significantly reduced. These results indicate that DusC is affected the most in the presence of paraquat, followed by DusB, while DusA is almost not influenced by the introduced redox changes. Again, this is consistent with LC–MS results on the respective knockout strains (Figure 5B).

### Differential enzyme activities of Dus enzymes are recapitulated *in vitro*

**Figure 7.**
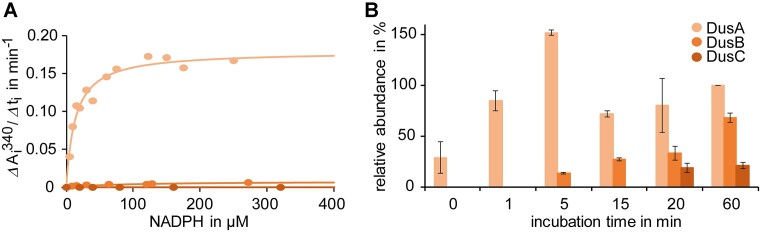
Kinetics of dihydrouridylation. (**A**) Activities of *E. coli* dihydrouridine synthases DusA (salmon), DusB (orange) and DusC (red). (**B**) *In vitro* assay of DusA, DusB and DusC. Dihydrouridine levels were detected by LC–MS/MS after different incubation times (0, 1, 5, 15, 20 and 60 min) of the respective enzyme with tRNA isolated from a triple knockout strain in the presence of NADPH. The dihydrouridine signals were normalized to the UV signal of adenosine and related to the normalized signal after an incubation with DusA for 60 min. The results are shown as average of biological duplicates; standard deviations are indicated as error bars.

In order to elucidate the molecular basis for the varying sensitivity of dihydrouridylation sites to paraquat *in vivo*, we conducted a comprehensive characterization of the redox reactivity of recombinant *E. coli* Dus enzymes *in vitro*. The three Dus enzymes, expressed and purified to homogeneity, exhibited non-covalently bound flavin, associated with the apoproteins, consistent with the expected flavoprotein nature of these enzymes within this class. The NADPH oxidase activity of the three Dus enzymes was quantified spectrophotometrically by monitoring NADPH consumption at 340 nm under aerobic conditions (Figure 7A). Intriguingly, the kinetic analyses revealed that DusA efficiently oxidized NADPH with an efficiency constant of 2.8 × 10^−2^ μM^−1^ s^−1^ (*k*_cat_ ∼ 0.48 ± 0.072 s^−1^, *K*_m_ ∼ 17 ± 3 μM), in contrast to DusB, which oxidizes NADPH more slowly with a *k*_cat_ ∼ 0.011 ± 0.003 s^−1^. The determination of a precise *K*_m_ value for NADPH was challenging due to the sluggish kinetics. Notably, DusC did not exhibit detectable NADPH oxidase activity, likely attributable to the exceedingly slow oxidation reaction of NADPH by FMN.

Subsequently, we sought to explore the impact of NADPH-dependent activity of Dus enzymes on tRNA dihydrouridylation. To this end, we quantified the *in vitro* dihydrouridylation of tRNAs over time (Figure 7B). Non-dihydrouridylated total tRNA (isolated from *ΔdusA,B,C*) was incubated with DusA, DusB or DusC for different incubation times, and resulting dihydrouridine levels were determined by LC–MS/MS. Dihydrouridylation by DusA was very fast, as dihydrouridine modification was already detected directly after the addition of DusA, and after an incubation of only 1 min D levels were close to saturation. In stark contrast, DusB-dependent D signals were detected only after 5 min, and DusC activity was weakest, with D levels reaching detection levels only after 20 min. Importantly, this shows that the DusC enzyme preparation was active despite its inactivity in the NADPH oxidation level (Figure 7A).

These findings establish a hierarchy in the efficiency of tRNA dihydrouridylation by Dus enzymes, with the order being DusA > DusB > DusC. This observed ranking is strongly indicative of variations in the redox reaction of NADPH with the flavin among the Dus enzymes, suggesting a crucial role of this redox activity in influencing their respective dihydrouridylation efficiencies.

## Discussion

Based on the *in vitro* requirement for NADPH as a reducing agent for dihydrouridine modifications in tRNAs, we hypothesized that cellular D levels might depend on the redox state as defined by the ratio of NADPH/NADP^+^. We therefore decided to manipulate the redox state of the cell by means of paraquat and to monitor the effect of redox changes on the dihydrouridine modification level. According to the literature, the bipyridinium salt paraquat unbalances the cellular redox system, leading to changes in NAD(P)H/NAD(P)^+^ ratios and might result in the production of reactive oxygen species. Within the so-called redox cycle, oxygen and reactive oxygen species oxidize the paraquat radical, resulting in its regeneration. NAD(P)H oxidases might serve as inducer of these redox cycles (45–47), indicating that the redox equivalents NADH and NADPH are involved in this process. Paraquat supplementation in a pilot experiment led to a dose-dependent reduction of growth, which we monitored by turbidity measurements at 600 nm. While detailed toxicity assessment by quantitative determination of colony-forming units might be better suited to characterize a detailed dose–response relation, our approach identified two paraquat concentrations whose effects could later be reenacted in conditions including oxygen control.

We indeed observed a reduction of D levels as a result of the paraquat treatment, in line with the said hypothesis. Importantly, the effect was distinct under aerobic conditions but absent under anaerobic conditions, where no significant difference was detected after paraquat supplementation. Analysis of NADPH levels by LC–MS, in combination with the requirements for oxygen and paraquat, confirmed that paraquat reduces NADPH levels, and that regeneration of paraquat, presumably as a result of the redox cycling with oxygen, increases the consumption of intracellular NADPH.

A most striking observation was the differential sensitivity of the Dus enzymes towards paraquat, revealed by two complementary methods. While LC–MS/MS allowed a precise quantitative assessment of D levels, sequence-specific information, and therefore information on differential activity of Dus enzymes, was missing. Overcoming this obstacle, we showed that AAS provides semi-quantitative results, including sequence information. This allowed the biosynthesis of distinct D residues in the various tRNAs to be directly attributed to catalytic functions of DusA, DusB and DusC. We could furthermore derive a detailed picture of the impact of paraquat on the global as well as local D synthesis. While several of our findings could be derived from studies on the *E. coli* wild-type strain, it is reassuring to observe that the results on the various knockout strains corroborate our findings. For example, the negative effect of paraquat on D synthesis was strongest in tRNA isolated from the DusA knockout strain, in which DusB and DusC are the active enzymes. Results from LC–MS and AAS obtained from all strains agree that, under the applied conditions, paraquat does not appear to affect DusA. The relative sensitivity of DusB and DusC is most plausibly explained by a paraquat-dependent drop of intracellular NADPH availability. This interpretation is strongly supported by *in vitro* kinetics. The kinetic analysis underscores DusA as the more efficient enzyme compared to DusB and DusC, attributed primarily to their distinct redox reactions with NADPH and their respective FMN coenzymes, with efficiency decreasing from DusA over DusB to DusC. These kinetic parameters faithfully mirror the relative *in vivo* activities of the enzymes. Consequently, these data offer a plausible explanation for the extent to which the reduced *in vivo* levels of NADPH impede dihydrouridine synthesis by DusB and DusC. To date, we lack both biochemical and structural information crucial for pinpointing the NADPH binding site in the Dus enzymes. However, based on our kinetic characterizations, DusA more efficiently binds and oxidizes NADPH than DusB, with DusC showing only minimal, yet still measurable activity. This leads to an intriguing hypothesis, namely that DusB and DusC may rely on co-actors aiding in the redox reaction *in vivo*, possibly akin to the biosynthesis of mcm^5^s^2^U34, which is known to necessitate the NADH/Cbr1/Dph3 system as an electron donor (55).

We extend our investigations to determine how far D levels affect tRNAs that are actively involved in translation. Comparison of D levels from tRNAs isolated from polysomes was used as a proxy to reflect functional tRNA populations. However, neither under normal growth conditions nor under paraquat treatment did we obtain any tangible evidence that would point out which exact interaction of tRNA in the translation apparatus might be affected by D. Noteworthy are two different interesting observations made on polysomes. The initiator tRNA^fMet^, isolated from DusA knockout and triple knockout strains, was found to be dihydrouridylated to a higher extent in the polysomal fraction (F3) (Figure 6A and Supplementary Figures S3 and S4), leading us to speculate that dihydrouridylation of the tRNA^fMet^ may be important for its binding to the ribosome. Further, there is one set of conditions, namely 0.3 mM paraquat treatment of the DusA knockout strain, where the DusC-dependent D residues at position 16 are enriched in the polysomal (F3) fraction, which was observed in two out of three replicates (bottom of Figure 6A and Supplementary Figure S3). Given that these data are too tentative to derive any solid conclusions, we speculate that at least certain D residues might modulate tRNA participation in protein biosynthesis under *some* conditions, which are yet to be found. Given that D modulates tRNA thermal stability, we are inclined to continue our investigations at lower growth temperature.

## Supplementary Material

gkae964_Supplemental_Files

## Data Availability

Sequencing data (AAS) are available at the European Nucleotide Archive (ENA) with the accession number PRJEB73750. LC–MS data are available as Supplementary Table S1. All other data needed to evaluate the conclusions of the paper are present in the paper and Supplementary Data.
