# Supplementary material for: Differential redox sensitivity of tRNA dihydrouridylation
Source: Nucleic Acids Res. 2024 Oct 26;52(21):12784–97. doi: 10.1093/nar/gkae964 (PMC11602153; doi:10.1093/nar/gkae964)
Supplement: gkae964_Supplemental_Files [file gkae964_supplemental_files.zip › Kilz NAR 2024 Supplementary Data.docx]

**Supplementary Data**

**Differential redox sensitivity of tRNA dihydrouridylation**

Lea-Marie Kilz^1^, Simone Zimmermann^1^, Virginie Marchand^2,3^, Valérie Bourguignon^2,3^, Claudia Sudol^4,5^, Damien Brégeon^4^, Djemel Hamdane^5^, Yuri Motorin^2,3^, Mark Helm^1,^*

^1^ Institute of Pharmaceutical and Biomedical Sciences, Johannes Gutenberg-University Mainz, 55128 Mainz, Germany

^2^ Université de Lorraine, CNRS, INSERM, UAR2008/US40 IBSLor, EpiRNA-Seq Core Facility, Nancy, F-54000, France

^3^ Université de Lorraine, CNRS, UMR7365 IMoPA, Nancy, F-54000, France

^4^ Sorbonne University, CNRS, Institute of Biology Paris Seine, Biology of Aging and Adaptation, Paris, 75252, France

^5^ Collège De France, Sorbonne Université, CNRS, Laboratoire de Chimie des Processus Biologiques, LCPB, 75231 Paris Cedex 05, France

* To whom correspondence should be addressed. Tel: +49 (0) 6131 39 25731; Fax: +49 (0) 6131 39 20373; Email: mhelm@uni-mainz.de


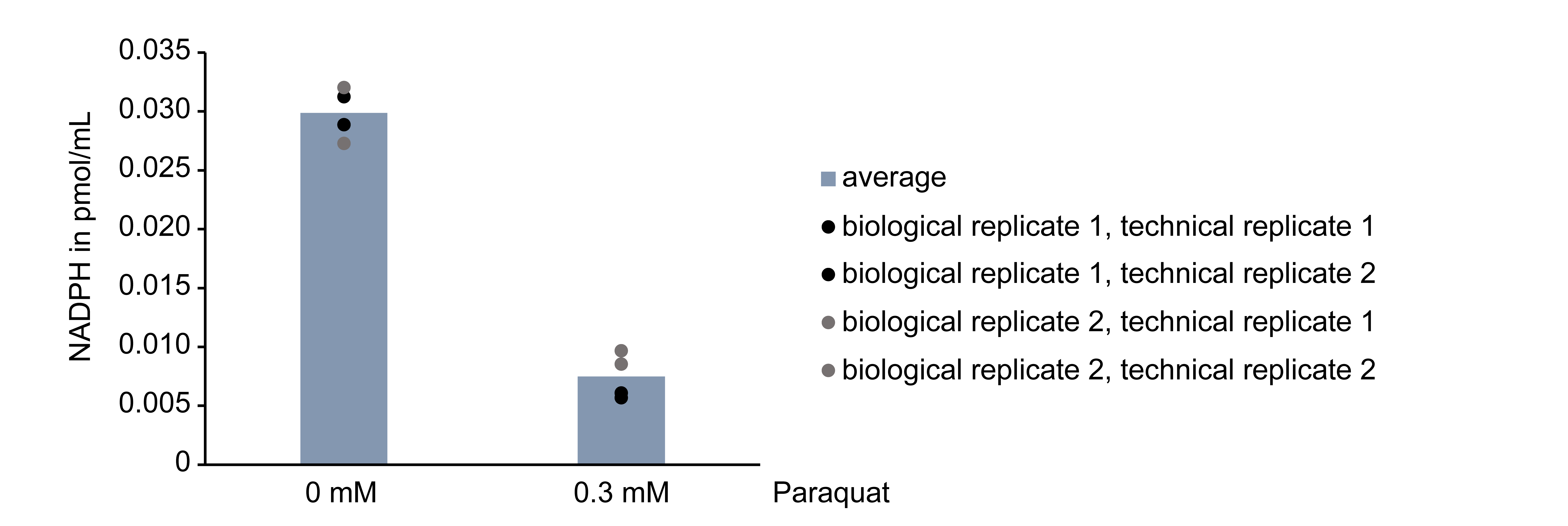


**Figure S1: NADPH level in *E. coli* cultures.** LC-MS/MS analysis of NADPH extracted from untreated and treated (0.3 mM paraquat) wildtype *E. coli* cultures grown under aerobic conditions. The NADPH levels were calculated relative to biomass, given as culture volume in mL at an optical density of 1. Results are shown as average of biological duplicates with technical duplicates for each culture (blue). The data points of each technical replicate are depicted as dots in black (biological replicate 1) and grey (biological replicate 2).


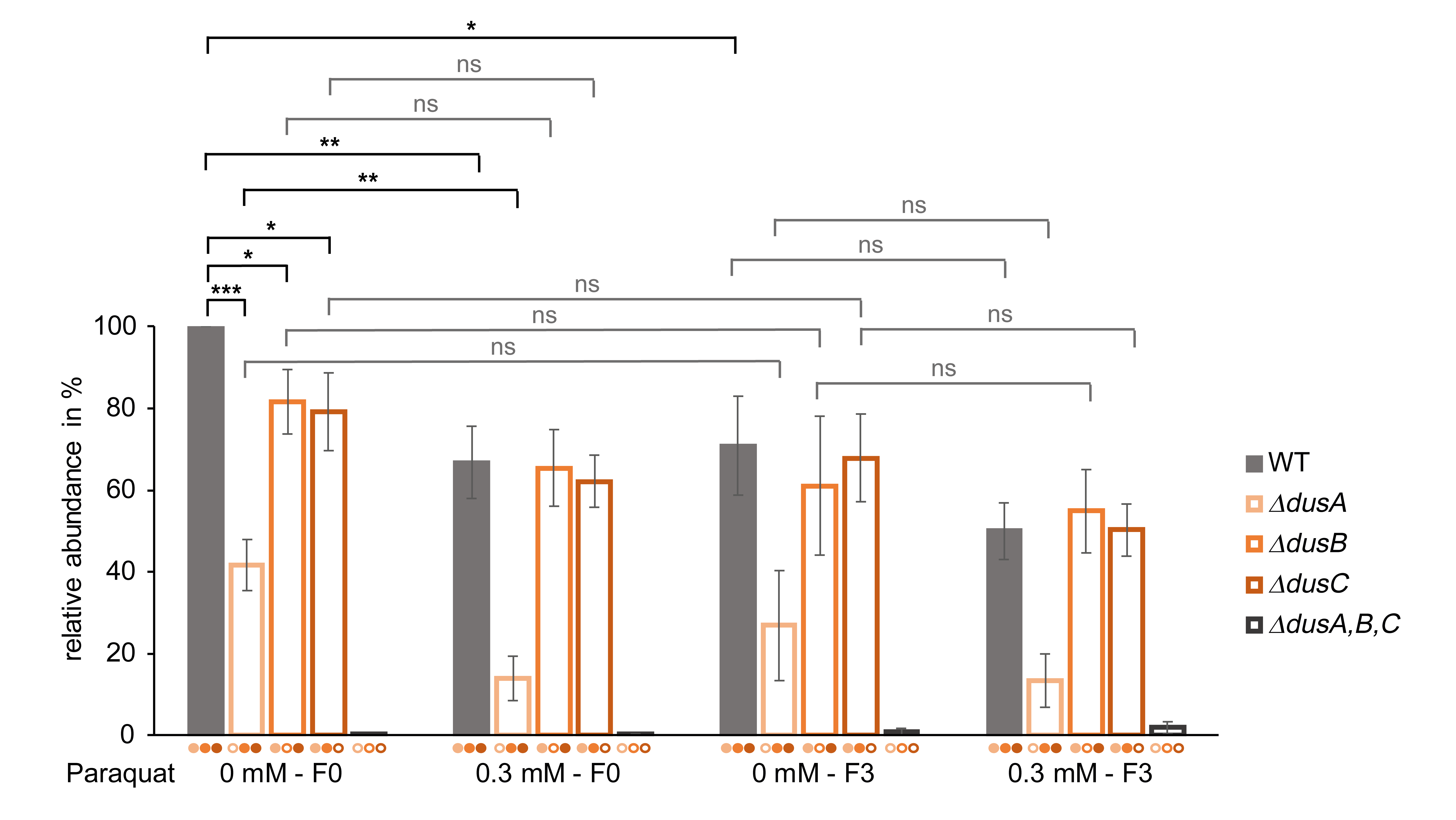


**Figure S2: Polysome preparations of *E. coli* cultures treated with paraquat and analysis of isolated tRNA.** Replicate of Figure 5B including significances of the LC-MS/MS analysis of dihydrouridine in tRNA isolated from free tRNA fraction (F0) and polysomal fraction (F3) of treated (0.3 mM paraquat) and untreated wildtype (grey) and knockout cultures (*ΔdusA*: salmon, *ΔdusB*: orange, *ΔdusC*: red). Filled circles indicate active enzymes and empty circles mark the knockout of the enzyme. LC-MS/MS signals were normalised to the UV signal of adenosine and the normalised signal of D in tRNA isolated from free RNA fraction of untreated wildtype culture (WT 0 mM F0) was set to 100%. Experiments were performed in biological triplicates and results are shown as average, the standard deviations are depicted as error bars. Statistical significances were determined using a two-tailed Student’s t-test (***: p < 0.001, **: p < 0.01, *: p < 0.05, ns: not significant). Significant changes are highlighted in black, whereas non-significant changes are depicted in grey.

**

**

**Figure S3: AlkAniline Sequencing of tRNA isolated from *E. coli* polysome preparations.** Detection of dihydrouridine modification sites in tRNAs isolated from free RNA fraction (F0) and polysomal fraction (F3) that were obtained from treated (0.3 mM paraquat) and untreated *E. coli* wildtype (WT) and single knockout (*ΔdusA*, *ΔdusB* and *ΔdusC*) cultures. Results of one biological replicate are shown. Filled circles mark the active Dus enzymes, while empty circles indicate that the enzyme is knocked-out. The different tRNAs and respective positions of dihydrouridine modifications are indicated on the right. Results are shown as Stop Ratio.

**

**

**Figure S4: AlkAniline Sequencing of tRNA isolated from *E. coli* polysome preparations.** Detection of dihydrouridine modification sites in tRNAs isolated from free RNA fraction (F0) and polysomal fraction (F3) that were obtained from treated (0.3 mM paraquat) and untreated *E. coli* wildtype (WT) and single knockout (*ΔdusA*, *ΔdusB* and *ΔdusC*) cultures. Results of one biological replicate are shown. Filled circles mark the active Dus enzymes, while empty circles indicate that the enzyme is knocked-out. The different tRNAs and respective positions of dihydrouridine modifications are indicated on the right. Results are shown as Stop Ratio.
